# Analysis of Changes in the Selected Nutritional Parameters of Patients within a Year from the Admission to the Enteral Nutrition Clinic

**DOI:** 10.3390/nu15081803

**Published:** 2023-04-07

**Authors:** Mariola Konecka, Magdalena Kuczyńska, Daria Schneider-Matyka, Marzanna Stanisławska, Elżbieta Grochans, Magdalena Kamińska

**Affiliations:** 1 Subdepartment of Long-Term Care and Palliative Medicine, Department of Social Medicine, Pomeranian Medical University in Szczecin, Żołnierska Str. 48, 71-210 Szczecin, Poland; 2 Department of Nursing, Pomeranian Medical University in Szczecin, Żołnierska Str. 48, 71-210 Szczecin, Poland

**Keywords:** malnutrition, nutrition therapy, SGA, NRS

## Abstract

(1) The following research question was formulated: What are the relationships between enteral nutrition and selected anthropometric and blood biochemical parameters? The aim of this study was to provide an assessment of the nutritional status of patients within one year from their admission to the Enteral Nutrition Clinic. (2) The study group included 103 participants. For the purpose of analysing their nutritional status, the Subjective Global Assessment (SGA) and Nutritional Risk Score (NRS) scales were used, anthropometric measurements were taken, and blood laboratory tests were performed. The assessment of changes in the indicated parameters was conducted at three time intervals: upon admission (T_0_) and 6 and 12 months after admission (T_6_ and T_12_, respectively). (3) The study group showed a significant improvement in the circumference of their upper and lower limbs. Nutrition therapy had an effect on the levels of erythrocytes, iron concentration, the activity of liver enzymes, and C-reactive protein levels. (4) The enrolment of patients into the Nutritional Therapy Programme had a positive effect on the selected results. 1. Twelve months after the introduction of nutritional intervention, an increase in erythrocyte count was particularly marked, and there was a decrease in the CRP (C Reactive Protein) level as well as the activity of liver enzymes. There was no significant effect of enteral nutrition on albumin and protein values. 2. To ensure the greatest efficiency of enteral nutritional therapy, it is to be continued for more than six months. 3. Nutritional interventions resulted in a significant increase in upper and lower limb circumferences among the study group. 4. For the purpose of identifying patients at risk of malnutrition, medical personnel should systematically raise their qualifications, and educational measures on this issue should be implemented at the stage of medical training at medical universities.

## 1. Introduction

Proper nutrition is one of the crucial elements of maintaining optimal health. The provision of properly balanced nutritional products plays a special role in treatment and convalescence [1]. Underlying disease and comorbid conditions may contribute to decreasing the ability of an organism to ingest food independently, and this may result in malnutrition.

According to the European Society for Clinical Nutrition and Metabolism (ESPEN), malnutrition is a condition resulting from the lack of absorption or intake of nutrients, leading to changes in body composition and a reduction in the free fat mass (FFM) and body cell mass (BCM) which may impair the physical and mental capabilities of an organism and have a negative effect on the treatment of an underlying disease [2,3].

The screening tests conducted by the British Association for Parenteral and Enteral Nutrition (BAPEN) demonstrated the occurrence of nutritional disorders in almost one third of emergency-admitted patients (28%), 22% of whom were at high risk and 6% of whom were at medium risk for nutritional disorders [3]. The factors associated with malnutrition were diseases of the digestive system—41%; neurological diseases—31%; and neoplastic diseases, all types—40%. A similar prevalence of nutritional disorders was identified in numerous countries in Europe as well as in America [3,4].

Upon diagnosis of malnutrition, nutritional treatment is to be implemented with the aim of maintaining, if possible, the physiological enteral route, thus preventing the atrophy and dysfunction of the digestive system [5,6].

The initiation of nutritional therapy is preceded by the analysis of the nutritional status of a patient, conducted with the use of standardised measurement scales such as the *Subjective Global Assessment* (SGA) or *Nutritional Risk Score* 2002 (NRS) in combination with a physical examination and blood chemistry results.

The anthropometric measurements include body weight, height, BMI and measurements of thigh circumference, arm circumference, abdomen circumference and skinfold thickness, which is particularly useful in the case of bedridden patients [3]. The measurement of arm circumference are taken with a measuring tape halfway between the coracoid process and the olecranon (*Midarm Circumference—MAC*). The most commonly determined biochemical parameters are: peripheral blood morphology, lipid profile and glucose. It is also advisable to determine the levels of albumin and/or short half-life proteins (e.g., prealbumin, transferrin), C-reactive protein (CRP), some vitamins and/or elements (potassium, magnesium, calcium, iron, phosphorus, zinc) [7]. At the assessment of malnutrition, special attention should be paid to particular biochemical parameters. Among proteins, albumin and transferrin, for example, play an important role in the assessment of malnutrition [3,7]. The significant role of the relationship between pro-inflammatory cytokines, e.g., IL-6, IL-10, or THF-α, and patient nutritional status is also noteworthy [8,9]. When interpreting albumin levels with respect to the nutritional status, it is assumed that a serum level below 3.5 g/dL is indicative of malnutrition, and a serum albumin level <2.1 marks a severe case of malnutrition [3,10]. The levels of transferrin, which is responsible for serum iron transport, also provide information about the severity of malnutrition. Anaemia is one of the earliest symptoms of chronic protein–calorie malnutrition. A normal lymphocyte count is over 1500 in one cubic millimetre of peripheral blood. A decreased count of below 800 may indicate severe nutritional and immune impairment. For the purpose of estimating the inflammatory process accompanying both the disease and malnutrition, an analysis of the C-reactive protein (CRP) in serum is used. An elevated level may be a symptom of ongoing inflammation. Additionally, systematic biochemical tests conducted on patients undergoing nutritional therapy include: the activity of liver enzymes, creatinine, urea, levels of electrolytes, calcium, phosphate, and magnesium [3,7].

Nutritional treatment involves the use of the so-called kitchen variety blended diet, which is implemented in patients with contraindications for the industrial diet. Another reason for this, especially in countries of a low economic level, could be the economic aspect of implementing the industrial diet [3]. The introduction of the so-called blenderized formulations (BFs) is worthy of consideration [11]. However, if a patient meets the criteria for an industrial diet, this treatment method is to be considered optimal. Pursuant to the European Directive 1999/21/EC, dated March 1999, the industrial diet is referred to as dietary foods for special medical purposes [3,12]. The characteristics of the industrial diet that are particularly crucial in nutritional therapy include the precisely determined nutritional value and standardised composition of dietary food in each container. This makes it possible to determine the form of protein—as a source of nitrogen, energy density, osmolality, content and type of lipids, and other elements of every kind—in such a diet which, in turn, allows the tailored selection to meet the current needs of a patient. In a long term, this contributes to an improvement in the patient’s nutritional status [8,13,14].

The aim of the present study was to analyse the nutritional status of patients within one year of their admission to the Enteral Nutrition Clinic. 

## 2. Materials and Methods

The study involved the analysis of the medical records of 103 patients enrolled in the Nutritional Therapy Programme conducted at the Enteral Nutrition Clinic at The West Pomeranian Hospice for Children and Adults. The study was retrospective in nature. The mean age of the patients was 60.67 ± 16.79.

The study data were pseudonymised by the data administrator, The West Pomeranian Hospice for Children and Adults. The study was approved by the Bioethics Committee of Pomeranian Medical University (KB-0012/40/01/19). The results presented in this article are only a fragment of an extensive study on the nutritional status of patients at the Enteral Nutrition Clinic. The data under analysis in the present study were obtained during scheduled medical/nursing visits. The study involved measurements taken at three time intervals: T_0_ (upon admission to the Nutritional Therapy Clinic), T_6M_ (six months from T_0_), and T_12M_ (twelve months from T_0_).

The patients included in the study were unable to take food orally, mostly due to complicated underlying disease: tumours in the oral cavity or dysphagia due to neurological reasons. The criteria for the admittance of patients to the Enteral Nutrition Clinic for nutritional therapy as well as for inclusion in the present study were based on the standards developed by the Polish Society for Parenteral and Enteral Nutrition and Metabolism. According to the standards, the patients were referred to the Enteral Nutrition Clinic due to an ICD-10 diagnosis (International Statistical Classification of Diseases and Related Health Problems ICD), had an artificial access percutaneous endoscopic gastrostomy (PEG), gastrostomy, or jejunostomy, and both the patient and the caregiver were residents of the West Pomeranian voivodeship. The anthropometric measurements taken into consideration in the present paper were the right upper limb circumference (RULC), right lower limb circumference (RLLC), waist circumference, height [cm], and body weight [kg]. Next, the body mass index (BMI) was calculated [kg/m^2^]. In women, the norm for arm circumference is 16–23 cm, whereas in men it is 18–25 cm. Values below the normal levels indicate malnutrition [15,16]. The literature on the subject lacks a norm with respect to thigh circumference.

During house visits, biological material was collected for biochemical analysis—venous blood was collected according to the procedures for collecting, storing, and transporting peripheral venous biological material. The procedure was performed by nurses employed at the Enteral Nutrition Clinic. The analysis of the material was conducted using commonly applied methods in the Diagnostyka Laboratory, West Pomeranian Voivodeship.

To assess the nutritional status of patients, a Nutritional Risk Score (NRS 2002) was used. Values ≥3 indicated the need to implement nutritional treatment [3,7,17]. The assessment of nutrition was additionally extended using the Subjective Global Assessment (SGA). Next, the scores were summed, providing grounds for the subjective analysis of nutritional status, i.e., a correct nutritional status, suspected malnutrition or moderate malnutrition, cachexia, and a high risk of malnutrition [3,7,17]. In conclusion, the malnutrition of patients qualified for treatment in the Enteral Nutrition Clinic included in the present study was assessed by means of the results of the NRS and SGA scales and anthropometric measurements.

### Statistical Analysis

The variables were characterised using parameters of descriptive statistics selected depending on the type of measurement scale. For the variables expressed on the quantitative scale, the following were determined: measures of central tendency (M—mean; Mdn—median), measures of variability (SD—standard deviation; CV—coefficient of variation), and measures of location (IQR—interquartile range; minimum and maximum value of the measurement). For the variables expressed on the qualitative and ordinal scale, counts (N) and frequency (%) were determined. The following mathematical statistics methods were applied: a one-way analysis of variance (ANOVA), with the estimation of η-square (η^2^) factor and Dunnet’s or Scheffe’s *post hoc* test; an ANOVA with repeated measures with the estimation of η-square (η^2^) factor; Friedman repeated measures analysis of variance by ranks with the estimation of Kendall conformity factor; McNemar–Bowker’s test (Bowker’s test for internal symmetry) for dependent samples.

For all analyses, the statistical significance level of 0.05 was assumed a priori. The calculations were performed using STATISTICAL v. 13.3 software (TIBCO Software Inc., Palo Alto, CA, USA).

## 3. Results

The majority of the study group were male (59.2%), married (44.7%), and residents of cities with a population of over 100 thousand residents (48.5%). Neoplastic disease was the underlying disease for most of the patients in the study group (44.7%), and malnutrition was the most common comorbidity (52.4%). Details are presented in Table 1.

### 3.1. SGA Scale, NRS Scale

The analysis of nutritional status in the study group, which was conducted using the SGA scale, did not reveal any statistically significant differences between the 3rd and 6th month after enrolment in the nutritional therapy programme (*p* > 0.05). Data are presented in Table 2.

Additionally, a comparison was made between the time intervals T_0_–T_12_ which showed no statistically significant differences (Table 3).

### 3.2. Laboratory Results

Table 4 presents the results of all laboratory tests performed in the study group at time intervals T_0_, T_6M_, and T_12M_. In a number of areas, there was a statistically significant improvement in the results when compared with the results recorded upon admission/enrolment to the Nutritional Treatment Programme. For morphology, the greatest effect was identified regarding erythrocytes (η^2^ = 0.204; *p* < 0.0001). The greatest change in erythrocyte level was observed at time intervals T_0_ vs. T_12M_ (M ± SD: 3.84 ± 0.58 vs. 4.2 ± 0.49; *p* < 0.001).

Within one year from the initiation of the treatment, a marked decrease was found in the CRP level (T_0_ 28.00 mg/L vs. T_12M_ 12.94 mg/L; *p* < 0.0001). The concentration of C-reactive protein CRP [mg/L] was statistically higher at T_0_ compared with T_6M_ and T_12M_ (M ± SD: 28 ± 28.16 vs. 12.97 ± 11.59; *p* < 0.001 and 28 ± 28.16 vs. 12.94 ± 14.7; *p* < 0.001, respectively).

A statistically significant decrease in activity of all three liver enzymes was identified: ALT (*p* < 0.0001)—the greatest effect of enteral nutrition was observed with respect to this parameter (effect size η^2^ = 0.096), AST (*p* < 0.0001), and ALP (*p* = 0.001). The mean ALT activity [U/L] recorded for the patients under analysis was statistically significantly higher at T_0_ when compared with the mean activity at T_6M_ (M ± SD: 29.9 ± 18.47 vs. 25.59 ± 10.93; *p* < 0.001) and at T_12M_ (M ± SD: 29.9 ± 18.47 vs. 24.19 ± 10.18; *p* < 0.001).

Within one year from the initiation of nutritional treatment, the glucose level was normalised—from 109.96 mg/dL at T_0_ to 95.30 at T_12M_ (*p* < 0.0001). The mean glucose [mg/dL] level determined for the patients was statistically significantly higher at T_0_ when compared with the mean levels at T_6M_ (M ± SD: 109.86 ± 29.39 vs. 97.49 ± 15.62; *p* < 0.001) and T_12M_ (M ± SD: 109.86 ± 29.39 vs. 95.3 ± 11.06; *p* < 0.001).

Out of all the laboratory tests under analysis in the present paper, the greatest effect (η^2^ = 494) was observed with respect to changes in iron levels: 30.70 µg/dL at T_0_ and 49.12 µg/dL at T_12M_. The mean level of iron [mmol/L] determined for the patients was statistically significantly lower at T_0_ in comparison with the mean levels at T_6M_ (M ± SD: 30.7 ± 13.94 vs. 41.79 ± 10.51; *p* < 0.001) and at T_12M_ (M ± SD: 30.7 ± 13.94 vs. 49.12 ± 15.01; *p* < 0.001).

It is noteworthy that within one year from the initiation of nutritional treatment, there were no statistically significant changes in albumin and total protein (*p* > 0.05). The levels were within the reference values.

### 3.3. Anthropometric Parameters

In a comparison of the anthropometric measurements taken during the study period, out of the circumference of the right upper limb, circumference of the right lower limb, waist circumference, and BMI, only for the circumference of the right upper limb—RULC (*p* = 0.020) and the circumference of the right lower limb (*p* = 0.013) were significant changes demonstrated. The study did not reveal a significant effect of enteral nutrition on waist circumference or BMI (Table 5).

## 4. Discussion

Despite ever more perfect methods and therapeutic means implemented in providing care to patients with an advanced pathological process, a properly balanced diet shows a significant effect on the patient’s health status. Nutrients in food contribute significantly to the optimisation of the blood biochemical parameters of patients, whereas nutrient deficiency is often responsible for worsening the condition of the patient. Presently, nutrition disorders are one of the most pressing medical and social issues [18]. As a condition, malnutrition stems from numerous causes of a health-related, social, and economic character. It can also be a complication of an underlying disease due to poor absorption or excessive loss of nutrients, as well as a combination of the above-mentioned factors [19].

In cases where providing food orally becomes impossible or ineffective due to the patient’s condition, the only route of administering nutrients is enteral nutrition therapy. It is provided in specialised facilities under the strict supervision of an enteral nutrition therapeutic team. One of the key elements of this therapy is the regular monitoring of the patient’s nutritional index, including a wide spectrum of tests such as questionnaires and physical examinations coupled with anthropometric assessments and laboratory tests. The results of the subsequent stages of treatment are evaluated and compared with respect to the previously obtained results, particularly with the initial results. This allows for an objective assessment of the nutrition parameters and further modification to meet the current nutrient demand of the patient. It is worth noting that the results of our own studies suggest that in order to achieve the highest efficiency of enteral nutrition, it is to be conducted over a period of time longer than six months. This assessment was analysed, and this study presents a fragment of the wider research. The review of the literature on enteral nutrition shows that the present study, which focused on the comparative analysis of selected nutritional parameters at three time intervals, is, in fact, a pioneer study. The authors believe that this analysis will be an inspiration for the nutrition therapy teams of other facilities worldwide to pursue the issue further. However, due to the lack of publications on this issue, it was impossible to compare the results of our own studies with those obtained by other authors, particularly with respect to the effect of enteral nutrition on biochemical and anthropometric values recorded after six and twelve months of treatment.

Our own studies show that half of the patients under study (50.5%) were diagnosed with cachexia, according to the SGA scale. This indicates a high risk related to nutrition disorders among patients with an underlying chronic disease. Furthermore, it proves that there are difficulties connected with the initial diagnostic process. The literature on the subject also shows that the issue is recognised not only in Poland. According to Keller, one of the problems with diagnosing malnutrition is the lack of a uniform definition and standards for the applied nutrition methods and malnutrition diagnosis [20]. The results by Kaiser et al. from 12 countries demonstrated that the frequency of malnutrition among the elderly was approx. 23%, with the frequency of malnutrition found in patients at rehabilitation facilities 50.5% higher; the results also showed the incidence in hospitalized patients was 38.7% [21].

Half of the patients under study (44.7%) were diagnosed with neoplastic disease. Similar results were obtained by Marley et al., who observed that approx. 50% of hospice patients suffer from malnutrition and weight loss [22]. The study by *Zhang* et al., conducted with the use of SGA, confirmed the relationship between the deteriorating health status of geriatric patients with neoplastic disease and the mortality rate [23]. Malnutrition, which was also assessed with SGA, was found to be very frequent (80.92%) in elderly patients with *colorectal cancer*, as presented by Wang et al. [24]. It was found that malnutrition often results in recurring infections and a significant decrease in the patent’s quality of life [25]. A number of tools for the assessment of nutritional status and conducting screening tests were developed, e.g., the Nutritional Risk Score 2002 (NRS-2002), Mini Nutritional Assessment (MNA), Malnutrition Universal Screening Tool (MUST), or the Subjective Global Assessment (SGA) [26,27]. The intended objectification of the assessment has not been reached yet, as the tools apply different criteria and cut-off points. These tools were developed for different purposes and populations, and therefore are not used as universal tools in different clinical situations. Owing to the complexity of the aforementioned methods, practitioners search for rapid and efficient laboratory methods, usually including serum biochemical tests conducted as a part of routine blood analysis for the purpose of identifying patients at risk of malnutrition [27]. The measurements were also taken into consideration in our own studies. Blood biomarkers, particularly albumins, are frequently used in the diagnosis of malnutrition in a clinical setting. The study by Krawczyk et al. indicated the usefulness of blood biomarkers in the assessment of nutritional status [28]. As a biomarker of malnutrition, hypoalbuminaemia is a factor for poor prognosis in the course of acute illnesses, as presented by Eckart et al. [29]. Additionally, a relationship between blood biomarkers and chronic conditions was demonstrated [30]. However, the literature on the subject is lacking in evidence-based clinical guidelines which would assist in the determination of biomarkers, taking into consideration the individual condition of a given patient, i.e., the clinical status, diagnosis, and often a broad-spectrum of comorbidities. As discussed by Hickson [31], for the assessment of malnutrition, particular attention should be paid to albumin, prealbumin, haemoglobin, total cholesterol, and total protein. Our own studies, which include the analysis of the factors as specified by Hickson, showed that all parameters were within the reference values with the exception of haemoglobin and erythrocytes, both of which were found to be below norm. The assessment of the aforementioned parameters six months after the initiation of treatment showed a slight increase, yet was still below the norm. After twelve months of treatment, the mean values of haemoglobin and erythrocytes were within the reference values. This is in line with the results by Barroso et al., who demonstrated an improvement in red blood cell values 32 weeks after the initiation of nutrition therapy [32].

The approach to diagnosing malnutrition in adults based on aetiology recognises the significance of inflammation [33]. An increase in the C-reactive protein to prealbumin ratio in patients in intensive care units was associated with increased mortality [34]. However, Hickson [31] claims that CRP, TLC, and WBC levels may only serve as indicators of inflammation and are not good indicators of malnutrition. In our own studies, the mean CRP value was above the reference values and amounted to 28 mg/L. The mean leukocyte levels in patients under study were within the normal range. When assessed six and twelve months after the initiation of the treatment, the aforementioned parameters were in the trend of changes. 

The routine measurement of the prealbumin level is considered to be a useful nutritional and prognostic indicator in malnutrition pathophysiology [35]. For decades, albumin has been determined as an indicator of malnutrition in patients in clinically stable conditions. Serum albumin levels decrease with age by approx. 0.1 g/L per year; however, age on its own is not a reason for hypoalbuminaemia. Studies, for example by Cobrerizo et al. [33], demonstrate that albumin is the most researched protein for the purpose of diagnosing malnutrition. In our own studies, serum total protein levels were determined. An upward trend was identified after twelve months—the mean albumin levels were within the normal range and amounted to 4.75 mg/dL.

Body mass index (BMI) is still considered vital in the assessment of nutritional status. It is assumed that a low BMI is a criterion for the diagnosis of malnutrition and is superior to biochemical blood analysis in identifying malnutrition with screening tests. It is vital to ensure the identification of all those at risk of malnutrition—a threshold of 23 kg/m^2^ is suggested for people aged 72, which is in line with the most recent ESPEN consensus recommending the use of <22 kg/m^2^ for people above 70 years of age [36]. Our own studies and the analysis of the selected anthropometric variables demonstrated that the BMI of patients at admission and at T_6M_ and T_12M_ were within the reference values. However, a significant increase was observed in the circumference of both the upper and the lower limb. Measurements of circumference of the upper limb, taken as part of the assessment of malnutrition among adults and children, are also recognised by other authors [37,38].

Despite a lack of studies by other authors on an assessment of the nutritional status of patients enrolled in an enteral nutritional therapy programme within one year from the initiation of treatment, it was possible to compare the results of our present study with recommendations and studies on similar topics. However, it must be emphasized that the generally understood nutritional interventions significantly increase the quality of life of patients and reduce mortality in given diseases [39], reduce readmission rate [40], and complement the implemented therapeutic process [41].

The nutritional indicators applied in the our present study, anthropometric measurements and biochemical blood tests, are recognised and widely used in the assessment of patients with malnutrition. The authors demonstrated a relationship between implementing enteral nutrition therapy and changes in the selected nutrition parameters. For some of the parameters, the change occurred only one year after the initiation of treatment. With respect to other aspects, such as protein and albumin levels, no significant effect was found. This indicates the need to continue further research on not only the relationship between malnutrition and selected parameters but also on their variability over months of nutritional treatment. 

### Limitations

It must be noted that a limitation of this study is the lack of a control group. With this in mind, further studies on the issue discussed should make an effort to include a control group in order to authenticate the final conclusions. 

## 5. Conclusions

1. Enrolment of patients in a Nutritional Therapy Programme had a positive effect on the selected results. Twelve months after the introduction of nutritional intervention, an increase in erythrocyte count was particularly marked, and there was a decrease in CRP level as well as the activity of liver enzymes. There was no significant effect of enteral nutrition on albumin and protein values.
2. To ensure the greatest efficiency of enteral nutritional therapy, it should be continued for more than six months.
3. Nutritional interventions resulted in a significant increase in upper and lower limb circumferences among the study group. The interventions taken in the course of the study had no effect on the BMI, which was within the reference values throughout the whole study period.
4. For the purpose of identifying patients at risk of malnutrition, medical personnel should systematically raise their qualifications, and educational measures on this issue should be implemented during the stage of medical training at medical universities.

## Figures and Tables

**Table 1:** Sociodemographic characteristics of the study group.

Variable	n	%
Sex		
● Female	42	40.8
● Male	61	59.2
Place of residence		
● Country	26	25.2
● City of up to 100 thousand residents	27	26.2
● City of more than 100 thousand residents	50	48.5
Marital status		
● Married	46	44.7
● Widowed	27	26.2
● Single	30	29.1
Diagnosis		
● Neoplastic disease	46	44.7
● Neurological disease	26	25.2
● Malnutrition	31	30.1
Comorbidities		
● Neoplastic disease	3	2.9
● Neurological disease	46	44.7
● Malnutrition	54	52.4

**Table 2:** Comparison of nutritional status, according to SGA, of the patients enrolled in the Nutritional Treatment Programme at admission, after 6 months, and after 12 months of treatment.

T_0_	T_6M_			χ^2^_(df = 2)_	*p* *
	1_Suspected Malnutrition	2_Malnutrition	3_Cachexia		
1_suspected malnutrition	9 (8.7%)	0 (0.0%)	0 (0.0%)	1.000	0.607
2_malnutrition	0 (0.0%)	41 (39.8%)	1 (1.0%)		
3_cachexia	1 (1.0%)	1 (1.0%)	50 (48.5%)		
**T_6M_**	**T_12M_**				
1_suspected malnutrition	10 (9.7%)	0 (0.0%)	0 (0.0%)	1.000	0.317
2_malnutrition	0 (0.0%)	41 (39.8%)	1 (1.0%)		
3_cachexia	0 (0.0%)	0 (0.0%)	51 (50.5%)		

* McNemar-Bowker’s test; *p*—*p*-value.

**Table 3:** Comparison of the risk related to nutritional status according to NRS of the patients enrolled in the Nutritional Treatment Programme at admission, after 6 and 12 months of the initiation of treatment.

Parameter	T_0_		T_6M_		T_12M_		χ^2^_(2)_	*p* *	W
	Mdn	IQR	Mdn	IQR	Mdn	IQR			
NRS	4.0	1.0	4.0	1.0	4.0	1.0	6.000	0.050	0.03

* Friedman’s ANOVA, Mdn—median, IQR—interquartile range, W—Kendall’s conformity factor.

**Table 4:** Biochemical test results at three time intervals—statistical differences.

Parameter	Norm	T_0_		T_6M_		T_12M_		F_(2, 101)_	*p* *	η^2^
		M	SD	M	SD	M	SD			
WBC (thousand/µL)	3.80–10.00	9.31	4.60	8.38	2.98	8.35	2.74	7.117	0.001	0.065
LYM (thousand/µL)	1.00–3.00	1.35	0.88	5.70	31.51	1.76	1.22	1.813	0.166	-
Hb (g/dL)	12.0–15.5	11.44	1.38	11.97	0.80	13.75	10.80	3.925	0.021	0.037
E (mln/µL)	4.20–5.60	3.84	0.58	3.90	0.45	4.20	0.49	26.186	0.000	0.204
PLT (thousand/µL)	180–430	314.79	106.64	295.43	74.48	283.63	74.21	8.047	0.000	0.073
Phosphorus (mmol/L)	0.85–1.85	1.34	0.77	1.75	1.19	1.58	0.68	7.935	0.000	0.072
Albumin (g/dL)	3.2–4.5	3.19	0.55	3.28	0.51	4.75	9.38	2.831	0.061	-
Total protein (g/dL)	6.0–8.0	6.02	0.53	6.10	0.48	6.65	3.46	2.601	0.077	-
CRP (mg/L)	0.00–5.00	28.00	28.16	12.97	11.59	12.94	14.70	45.311	0.000	0.308
Urea (mg/dL)	17.00–43.00	36.99	17.39	34.42	10.06	37.55	14.35	3.506	0.032	0.033
Creatinine (mg/dL)	0.70–1.20	0.68	0.30	1.24	4.76	070	0,.25	1.379	0.254	-
ALT (U/L)	0–38	29.90	18.47	25.59	1093	24.19	10.18	10.861	0.000	0.096
AST (U/L)	0–41	25.32	15.59	22.24	10.35	21.04	9.04	8.653	0.000	0.078
ALP (U/L)	40–130	102.77	55.92	88.39	32.35	91.94	44.96	7.019	0.001	0.064
Glucose (mg/dL)	60.00–99.00	109.86	29.39	97.49	15.62	95.30	11.06	34.245	0.000	0.251
Total cholesterol (mg/dL)	115.00–190.00	156.61	48.76	142.70	32.14	146.15	28.27	10.699	0.000	0.095
Triglycerides (mg/dL)	<150	114.23	64.39	110.19	43.78	111.71	35.45	0.701	0.497	-
Sodium (mmol/L)	135–145	135.47	5.10	145.37	98.81	137.35	4.04	0.892	0.412	-
Potassium (mmol/L)	3.50–5.50	4.51	0.59	4.23	0.52	5.71	13.57	1.040	0.355	-
Chlorides (mmol/L)	96–111	96.73	4.52	96.50	2.65	97.70	9.67	1.290	0.279	-
Iron (µg/dL)	59.00–158	30.70	13.94	41.79	10.51	49.12	15.01	100.301	0.000	0.496
Total calcium (mmol/L)	2.09–2.54	2.21	0.25	2.26	0.08	2.25	0.11	3.140	0.045	0.030
Magnesium (mmol/L)	0.70–0.9	0.92	0.41	0.97	0.28	1.08	0.40	11.481	0.000	0.101

* ANOVA with repeated measures; M—mean; SD—standard deviation; η^2^—effect size; WBC—leukocytes; LYM—lymphocytes; Hb—haemoglobin; E—erythrocytes; PLT—platelets; ALT—Alanine aminotransferase; AST—aspartate aminotransferase; ALP—alkaline phosphatase.

**Table 5:** Comparison of anthropometric parameters of the patients at admission and after 6 and 12 months of treatment.

Parameter	T_0_		T_6M_		T_12M_		F_(2, 101)_	*p* *	η^2^
	M	SD	M	SD	M	SD			
RULC (cm)	23.73	4.17	23.32	4.25	24.15	4.24	3.973	0.020	0.037
RLLC (cm)	39.12	6.03	37.96	6.89	39.15	6.60	4.406	0.013	0.037
Waist circumference (cm)	80.35	11.78	77.72	12.70	79.03	12.63	2.947	0.055	-
BMI	19.28	3.44	18.81	3.16	19.31	3.04	2.770	0.065	-

* ANOVA with repeated measures; M—mean; SD—standard deviation; η^2^—effect size; *p*—*p*-value; RULC—right upper limb; RLLC—right lower limb; BMI—a patient’s weight in kilograms divided by the square of height in meters.

## Data Availability

The datasets generated during and/or analysed during the current study may be made available by the corresponding author upon request.
